# Increasing trend of transmitted integrase inhibitor resistance in a cohort of antiretroviral therapy-naive people living with HIV

**DOI:** 10.1093/jac/dkad109

**Published:** 2023-04-08

**Authors:** Camilla Muccini, Laura Galli, Michela Sampaolo, Nicola Gianotti, Antonella Castagna, Diana Canetti

**Affiliations:** Department of Infectious Diseases, IRCCS San Raffaele Scientific Institute, Milan, Italy; Vita-Salute San Raffaele University, via Stamira D’Ancona 20, Milan 20127, Italy; Department of Infectious Diseases, IRCCS San Raffaele Scientific Institute, Milan, Italy; Laboratory of Microbiology and Virology, IRCCS San Raffaele Institute, Milano, Italy; Department of Infectious Diseases, IRCCS San Raffaele Scientific Institute, Milan, Italy; Department of Infectious Diseases, IRCCS San Raffaele Scientific Institute, Milan, Italy; Vita-Salute San Raffaele University, via Stamira D’Ancona 20, Milan 20127, Italy; Department of Infectious Diseases, IRCCS San Raffaele Scientific Institute, Milan, Italy

Drug regimens containing integrase strand transfer inhibitors (INSTIs) are widely recommended by international guidelines for ART-naive people living with HIV (PLWH).^1,2^ However, INSTI resistance mutations are uncommon and therefore their surveillance is not suggested before starting ART. The aim of the study was to evaluate the prevalence trend of transmitted INSTI drug resistance (DR) and to assess factors associated with transmitted INSTI resistance among ART-naive PLWH over the past decade.

We conducted a time-trend, single-centre study over the period 2009–19 on adult naive PLWH with a genotypic resistance test (GRT) performed before the ART initiation. GRT was determined by Sanger sequencing using an ABI PRISM 3130xl Genetic Analyzer (Applied Biosystems, Foster City, CA, USA); we included in the analysis the immediate pre-ART GRT available in people newly diagnosed with HIV infection.

The degree of resistance to each INSTI drug was calculated using the Genotypic Resistance Interpretation Algorithm of the Stanford HIV Drug Resistance Database Program (version 9.1.1) (https://hivdb.stanford.edu/hivdb/by-patterns/). The obtained scores were used to classify the presence of low-level, intermediate or high-level resistance to each INSTI drug and to the INSTI drug class. DR within the INSTI class was defined by the presence of at least low-level resistance to ≥1 drug of the INSTI class.

All the considered characteristics were measured at or within 180 days before the GRT date.

Results were reported as median (IQR) or frequency (%).

Characteristics of PLWH with at least low-level INSTI resistance or less than low-level INSTI resistance were compared using the chi-squared/Fisher’s exact test or the Wilcoxon rank-sum test.

The Cochran-Armitage test was used to assess linear trend in transmitted HIV INSTI DR prevalence over time.

A multivariable logistic regression model was fitted to determine factors associated with at least low-level transmitted INSTI DR; given the limited number of PLWH with transmitted INSTI DR. Three variables were included into the model: age; CD4 cell count at GRT; and HIV-1 subtype.

All the statistical tests were two-sided at 5% level and were performed using the SAS Software (release 9.4; SAS Institute, Cary, NC, USA).

Overall, 1223 ART-naive PLWH were evaluated: 1109 (90.7%) were male, 860 (70.3%) were MSM and the median age was 37 (IQR, 30–45) years. Italian origin was found in 578 (78.0%) and 971 (79.4%) were infected by HIV-1 subtype B. CRF01_AE (74.0%) was the most frequent HIV-1 non-B subtype observed. The median time to ART initiation was 2.9 (1.3–20.2) months; GRT was performed after a median time of 1.2 (0.5–2.9) months once diagnosed with HIV infection and CD4 cell count at GRT was 416 (259–58) cells/µL.

At least low-level and at least intermediate INSTI DR were reported in 18 (1.5%) and 6 (0.5%), respectively.

Major INSTI resistance mutations were uncommon; both E138K and R263K were found in 2 (0.2%) people, while each of G118R, G140S, Y143C, Y143R, S147G and Q148H in 1 (0.1%) (Table S1, available as Supplementary data at *JAC* Online). The most frequent accessory INSTI resistance mutations detected were T97A in 19 (1.6%) PLWH, L74I in 12 (1.0%), E157Q in 11 (0.9%) and G163R in 10 (0.8%).

Among PLWH with and without at least low-level INSTI DR, HIV-1 subtype non-B was found in 8 (44.4%) and in 244 (20.3%) (*P* = 0.018); G163K, an accessory INSTI mutation previously described as common in subtype non-B viruses from ART-naive patients, was detected in 7/8 (87.5%). Moreover, subtype non-B infection proportion increased from 5% in 2009 to 27.5% in 2019 (*P* for trend < 0.0001).

Overall, 1/10 (10.0%) of people with HIV-1 subtype B and 1/8 (12.5%) of people with HIV-1 subtype non-B with at least low-level INSTI DR had a strain resistant to more than one drug class. Overall, 51 (4.2%) PLWH had a virus resistant to NRTIs, 47 (3.8%) to NNRTIs and 22 (1.8%) to PIs.

Prevalence of at least low-level INSTI DR was anecdotal between 2009 and 2013 and then gradually raised from 1.3% in 2014 to 3.9% in 2019 (*P* for trend < 0.001) prevalence of at least intermediate INSTI DR increased from 0% in 2009 to 2% in 2019 (*P* for trend = 0.188). A significant increase over time in at least low-level DR prevalence has emerged to raltegravir and elvitegravir, not to dolutegravir and bictegravir (*P* for trend < 0.001), as shown in Figure 1(a and b).

**Figure 1.**
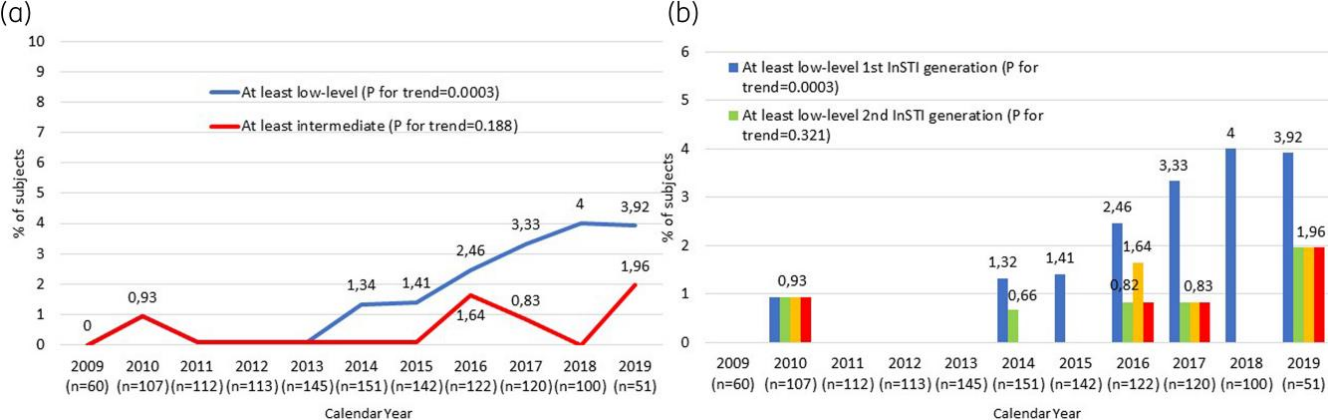
(a) Trend over time of at least low-level and at least intermediate transmitted DR prevalence. (b) Trend over time of transmitted DR prevalence according to first- and second-generation INSTIs. This figure appears in colour in the online version of *JAC* and in black and white in the print version of *JAC*.

At multivariable analysis, HIV-1 subtype non-B versus B was associated with INSTI resistance [adjusted OR 3.45 (95% CI = 1.36–8.85), *P* = 0.011], after adjusting for age and CD4 at baseline.

Over the decade 2009–19, the prevalence of at least low-level and intermediate INSTI DR among ART-naive PLWH with an available GRT performed before starting ART has mildly increased, together with the proportion of non-B subtype infections; nonetheless, major INSTI-resistance mutations were very uncommon.

INSTI polymorphisms that may decrease susceptibility to INSTIs are not infrequent;^3^ for instance, mutations like G163R/K are polymorphic in subtype F viruses but can also be non-polymorphic when considering other HIV-1 subtypes;^4^ however, if alone, they have little impact on INSTI activity.^5–7^

For this reason, HIV-1 subtype non-B has proved to be the only factor associated with INSTI resistance.

Current guidelines do not recommend INSTI resistance testing in naive PLWH, with the exception of a suspected exposure to INSTI-resistant strains.^8^ However, we have shown that first-generation INSTIs have a lower genetic barrier to resistance that could justify INSTI GRT implementation together with the evidence of the large use of INSTI regimens in ART-naive PLWH.

However, the small number of people with INSTI resistance mutations is a clear study limitation with an impact on the statistical power to detect significant effects.

In conclusion, the prevalence of at least low-level transmitted HIV-1 resistance to INSTIs is rare but has increased in recent years, mainly related to the first-generation INSTIs. Surveillance should still continue to monitor for future trends.

## Supplementary Material

dkad109_Supplementary_Data

## Supplementary data

Table S1 is available as Supplementary data at *JAC* Online.
