# Supplementary material for: Increasing trend of transmitted integrase inhibitor resistance in a cohort of antiretroviral therapy-naive people living with HIV
Source: J Antimicrob Chemother. 2023 Apr 8;78(5):1314–5. doi: 10.1093/jac/dkad109 (PMC10243965; doi:10.1093/jac/dkad109)
Supplement: dkad109_Supplementary_Data [file dkad109_supplementary_data.docx]

**Supplementary Table.** Resistance profile of people with at least low level resistance to INSTI.

|  | **Calendar year** | **Subtype** | **Major mutations** | **Minor mutations** | **Level of resistance to INSTI** |
| --- | --- | --- | --- | --- | --- |
| **ID 1** | 2014 | B |  | G163R | Low level R to RAL and EVG |
| **ID 2** | 2014 | B |  | S230R | Potential low level R to DTG and BIC and low level R to RAL and EVG |
| **ID 3** | 2015 | non-B |  | G163KR | Low level R to RAL and EVG |
| **ID 4** | 2016 | non-B |  | G163KR | Low level R to RAL and EVG |
| **ID 5** | 2017 | B | E138K | G163R, T97A, L741 | Potential low level R to DTG and BIC and intermediate R to RAL and EVG |
| **ID 6** | 2017 | B |  | G163R | Low level R to RAL and EVG |
| **ID 7** | 2018 | non-B |  | G163KR | Low level R to RAL and EVG |
| **ID 8** | 2017 | B |  | G163R | Low level R to RAL and EVG |
| **ID 9** | 2018 | non-B |  | G163KR | Low level R to RAL and EVG |
| **ID 10** | 2018 | non-B |  | G163KR | Low level R to RAL and EVG |
| **ID 11** | 2018 | non-B |  | G163KR | Low level R to RAL and EVG |
| **ID 12** | 2019 | B |  | G163R | Low level R to RAL and EVG |
| **ID 13** | 2010 | B | R263K |  | Low level R to BIC and RAL and intermediate level to DTG and EVG |
| **ID 14** | 2015 | B |  | G163R | Low level R to RAL and EVG |
| **ID 15** | 2016 | non-B | E138K, G140S, Y143C, Q148H | G163KR, T97A | High level R to DTG, BIC, RAL and EVG |
| **ID 16** | 2016 | B | S147G |  | High level R to EVG |
| **ID 17** | 2019 | B | G118R, 263K |  | Intermediate level to DTG and BIC and high level to RAL and EVG |
| **ID 18** | 2017 | non-B | G118R |  | Intermediate level to DTG and BIC and high level to RAL and EVG |

Abbreviation: ID, identifier; INSTI, integrase strand transfer inhibitors; R, resistance; RAL, raltegravir; EVG, elvitegravir; DTG, dolutegravir; BIC, bictegravir.
